# Assessing postnatal care for newborns in Sub-Saharan Africa: A multinational analysis

**DOI:** 10.1371/journal.pone.0298459

**Published:** 2024-02-15

**Authors:** Habitu Birhan Eshetu, Fantu Mamo Aragaw, Wubshet Debebe Negash, Tadele Biresaw Belachew, Desale Bihonegn Asmamaw, Abiyu Abadi Tareke, Melaku Hunie Asratie

**Affiliations:** 1 Department of Health Promotion and Health Behavior, Institute of Public Health, College of Medicine and Health Sciences, University of Gondar, Gondar, Ethiopia; 2 Department of Epidemiology and Biostatistics, Institute of Public Health, College of Medicine and Health Sciences, University of Gondar, Gondar, Ethiopia; 3 Department of Health Systems and Policy, Institute of Public Health, College of Medicine and Health Sciences, University of Gondar, Gondar, Ethiopia; 4 Department of Reproductive Health, Institute of Public Health, College of Medicine and Health Sciences, University of Gondar, Gondar, Ethiopia; 5 Amref Health Africa in Ethiopia, SLL Project, COVID-19 Vaccine /EPI Technical Assistant at West Gondar, Addis Ababa, Ethiopia; 6 Department of Women’s and Family Health, School of Midwifery, College of Medicine and Health Sciences, University of Gondar, Gondar, Ethiopia; Nurses’ and Midwives Training College, GHANA

## Abstract

**Background:**

No doubt providing optimal postnatal care (PNC) prevents both maternal and neonatal deaths, in addition to the prevention of long-term complications. Sub-Saharan Africa (SSA) had the highest neonatal mortality rate, despite this adequate content of PNC for the newborn is not explored in SSA, therefore, it is important to identify the factors affecting adequate content of PNC for the newborn in the region. This may assist the program and policymakers to give an intervention based on the findings of the study.

**Methods:**

A secondary data analysis was performed using 21 SSA countries’ Demographic and Health Surveys. A total weighted sample of 105,904 respondents were included in this study. A multilevel binary logistic regression model was fitted. The odds ratios along with the 95% confidence interval were generated to determine the individual and community-level factors of adequate PNC for the newborn. A p-value less than 0.05 was declared as statistical significance.

**Results:**

Adequate PNC for newborns in sub-Saharan Africa was 23.51% (95% CI: 23.26, 23.77). Mothers age ≥ 35(AOR  =  1.21,95% CI: 1.06,1.16), mothers’ primary education (AOR  = 1.18, 95% CI: 1.13, 1.23), secondary education (AOR  = 1.58, 95% CI:1.51,1.66), higher education (AOR  = 1.61,95% CI:1.49,1.75), rich wealth status (AOR  = 1.05,95% CI = 1.01,1.10), ANC visits 1–7 (AOR  = 1.61,95% CI:1.51, 1.73), antenatal care (ANC) visit 8 and above (AOR  = 2.54,95% CI: 2.32, 2.77), health facility delivery (AOR  = 4.37, 95% CI:4.16,4.58), lived in east (AOR = 0.23,95% CI = (0.20,0.26), central(AOR = 0.21,95% CI = 0.19,0.24), west African sub-regions (AOR = 0.23,95% CI = 0.21, 0.27), Urban dwellers (AOR = 1.22,95% CI: 1.17,1.27), and low community poverty (AOR = 1.21 (95% CI = 1.11,1.31) were associated with adequate content of PNC for the newborn.

**Conclusion:**

The finding of this study showed that the overall prevalence of adequate content of PNC for a newborn in SSA countries was low. The low prevalence of adequate content of postnatal care for newborns in SSA countries is a concerning issue that requires immediate attention. Age of the respondents, level of education, wealth status, ANC visits, place of delivery, residence, community-level poverty, and sub-region of SSA were the individual-level and the community-level variables significantly associated with adequate PNC for the newborn. Strategies should focus on increasing access to antenatal care services, particularly for vulnerable populations, such as younger mothers, those with lower education levels, and individuals residing in impoverished communities to improve PNC for the newborn.

## Background

Postnatal Care (PNC) is one of the care packages that comprise the worldwide continuum of care for mothers and newborns [1]. Maternal and child mortality rates are widely used to judge a country’s health system’s success. However, 2.4 million children died in their first month of birth worldwide in 2020. Over 6700 newborns die every day, accounting for 47% of all child mortality under the age of five [2]. Most neonatal deaths (75%) occur during the first week of life, and in 2019, about 1 million newborns died within the first 24 hours [2, 3].

Sub-Saharan Africa (SSA) had the highest neonatal mortality rate (27 deaths per 1000 live births) in 2020, followed by Central and Southern Asia and the Middle East (23 deaths per 1000 live births). A child born in SSA or Southern Asia is 10 times more likely to die in the first month of life than a child born in a high-income nation. The majority of neonatal deaths in impoverished nations are caused by childbirth, intrapartum, and inadequate immediate infant care practices [2, 4, 5].

The postpartum period is critical for both the mother’s and newborn child’s health and survival [6, 7]. It is suggested that women who give birth in a healthcare facility with a trained attendant get prompt PNC and stay at the facility for at least 24 hours in the case of an uncomplicated birth [8]. However, it has been observed that even when women give birth in a healthcare facility, PNC may not be included because women may only be there for a few hours [9]. Only 13% of the 48% of women in SSA who give birth without a trained birth attendant receive a PNC visit [10].

The international community established a sustainable development goal (SDG) to reduce neonatal and under-five mortality rates to 12 and 25 deaths per 1000 live births, respectively, by 2030 [11, 12]. Low-income countries, such as SSA, are falling behind in meeting the maternal and newborn health targets established under the SDG agenda [13].

Different studies show that neonatal deaths can easily be prevented and avoided in developing countries with simple, low-cost, and short-term newborn care [14–20]. Adequate content of PNC for the newborn defined in terms of contents that a newborn utilize included: having the cord examined, having the temperature of the baby measured, counseling on newborn danger signs, counseling on breastfeeding, and having had an observed breastfeeding session [7, 21].

There are no comprehensive studies regarding the content of PNC for a newborn in SSA though one single study has been conducted in Rwanda [7], but it does not take into account the hierarchical nature of the Demographic health survey (DHS). Therefore, this study aimed to assess the prevalence of adequate content of PNC for newborns in SSA countries using the recent DHS. A regional comparison is necessary to achieve current global initiative agendas such as the SDGs. Furthermore, the information derived from this study will provide invaluable insight into sub-regional adequate content of PNC for newborns. This study may also support policymakers, non-governmental organizations (NGOs), other global organizations, and researchers in identifying the factors in the African region that influence adequate content of PNC in order to provide rapid interventional measures and resource allocation to enhance their utilization of postnatal newborn care.

## Methods

### Study design and data source

This study used secondary data cross-sectional household data for women collected from 2015 to 2021 among 21 SSA countries demographic health surveys, namely, Burundi, Ethiopia, Malawi, Rwanda, Tanzania, Uganda, Zambia, Zimbabwe, Madagascar, Angola, Cameroon, Benin, Gambia, Guinea, Liberia, Mali, Mauritania, Nigeria, Senegal, Sierra Leone, and South Africa. We retrieved the data for this study from the DHS website www.dhsprogram.com after the request to access the data and downloading was allowed. In this study, we combined these datasets in order to generate large datasets representing different SSA countries and generalizing PNC for newborns in the countries. DHS is a nationally representative household survey that collects data on a broad range of health indicators like mortality, morbidity, fertility, family planning, and maternal and child health [21]. In this study, we used the child record datasets (KR file), and extracted the outcome and independent variables. A two-stage stratified selection procedure was used to identify study participants. In the first step, enumeration areas (EAs) were chosen at random, while households were chosen in the second stage. A total weighted sample of 105,904 respondents with their respective children were included in the study (Table 1).

**Table 1:** Sample size for adequate content of PNC for the newborn in SSA countries for each country, 2023.

Regions	Country	Year of survey		Weighted sample (n)	Weighted sample (%)
East Africa countries	Burundi	2016/17		5412	5.11
	Ethiopia	2016		4308	4.07
	Malawi	2015/16		6693	6.32
	Rwanda	2019/20		6471	6.11
	Tanzania	2015/16		4167	3.93
	Uganda	2016		5901	5.57
	Zambia	2018		3905	3.69
	Zimbabwe	2015		2454	2.32
	Madagascar	2021		9794	9.25
Central Africa countries	Angola	2015/16		5405	5.10
	Cameroon	2018		3924	3.70
West Africa countries	Benin	2017/18		5502	5.20
	Gambia	2019/20		3129	2.95
	Guinea	2018		3026	2.86
	Liberia	2019/20		2096	1.98
	Mali	2018		4150	3.92
	Mauritania	2021		8970	8.47
	Nigeria	2018		12935	12.21
	Senegal	2019		2327	2.20
	Sierra Leone	2019		3950	3.73
Southern Africa	South Africa	2016		1386	1.31
Total sample size				105904	100

### Populations

All newborns during the first 2 days after the birth, two years preceding the survey year across 21 SSA countries were our source population, while newborns in the selected Enumeration Areas (EAs) or primary sampling units of the survey clusters were our study populations. Those mothers who were either permanent residents or guests who slept in the selected residences with their newborns the night before the survey were eligible to be questioned [22]. Furthermore, from the included DHS data set, newborns having the missing value of the outcome variable were excluded based on the DHS guideline.

### Variables and data collection procedure

#### Dependent variable

The dependent variable was adequate content of PNC for the newborn. Based on the world health organization (WHO) recommendations [23] and the availability of data in the DHS dataset, which was interviewed two years preceding the survey [21]. Adequate content of PNC for the newborn was considered when a newborn was able to have received all the five PNC contents that included: having the cord examined, having the temperature of the baby measured, counseling on newborn danger signs, counseling on breastfeeding, and having had an observed breastfeeding session [7, 21].

#### Individual level variable

Several individual-level variables were included, age (15–24, 25–34, and 35–49 years), media exposure (yes/no), respondent currently working (yes/no), education level (no education, primary, secondary and higher education), wealth index generated using household asset data and by Principal Component Analysis (PCA) to classify the respondents into wealth quintiles (poor, middle, and rich), the number of ANC visit (0, 1–7, 8 and above), and child sex (male, female).

#### Community level variables

Since the DHS collects the data at the individual level except for residency and distance from the health facility, in this study, the rest of the community-level variables were included in the analysis by generating from the individual level variables, which includes community poverty level, community media exposure, and sub-regions of SSA. The community-level poverty was determined by the proportion of households in the poorest and, poorer quintiles obtained from the wealth index results. Categorized as low if the proportion of household which is from households belonging to the categories of poor was less than 50% and categorized as high if the proportion was greater than 50%. Community-level media exposure was created from the respondents’ exposure to newspaper/magazines, radio, and television after merging and recoding as Yes/No, and the results were classified as low (below the median) and high (above the median) if the respondents had exposure to at least one medium based on the median value.

### Data analysis

Stata version 14 statistical software was used for coding and data analysis. The data were adjusted and weighted throughout the analysis to ensure the DHS sample’s representativeness and to get credible estimates and standard errors prior to data analysis. We used women’s individual sample weight (V005/1000000) for this study to account for the hierarchical nature of the DHS data [22].

Multilevel modeling, also known as hierarchical linear modeling or mixed-effects modeling, is a statistical technique used to analyze data that has a hierarchical or nested structure. It is particularly useful when dealing with data that has multiple levels of analysis, such as individuals nested within groups or repeated measures nested within individuals [24]. In our analysis, DHS datasets have hierarchical data structures with individuals nested under geographical clusters (primary sampling units) and newborns were nested inside a cluster. This may affect standard logistic regression model assumptions such as equal variance and independence assumptions. Thus, in this study, four models were fitted: the empty model, which did not have explanatory variables, model I, which contained individual-level factors, model II, which contained community-level factors, and model III, which contained both individual and community-level variables. Because the models were nested, the Intra-class Correlation Coefficient (ICC), Median Odds Ratio (MOR), and Likelihood Ratio test (LLR) values, as well as the deviation (-2LLR), were utilized for model comparison and fitness, respectively. Model III was chosen as the best-fitting model because of its low deviation compared to other models.

The outcome variables’ random effects or measures of variation were estimated using the median odds ratio (MOR), Proportional Change in Variance (PCV), and Intra Class Correlation Coefficient (ICC). Taking clusters as a random variable, the MOR is defined as the median value of the odds ratio between the area at the highest risk and the clusters at the lowest risk clusters when randomly picking out two clusters, ${{MOR=e}^{0.95}}^{\sqrt{VA}}$. While, the ICC tells the variation of adequate PNC for the newborn between clusters, and is calculated as; $ICC=\frac{VA}{VA+3.29}*100\%$. Furthermore, the PCV shows the variation in the prevalence of adequate PNC for the newborn explained by factors and calculated as; $PCV=\frac{Vnull-VA}{V\;null}*100\%$ where; Vnull = variance of the empty model, and VA = area/cluster level variance [25, 26]. Finally adjusted odds ratios with 95% confidence intervals and a p-value of less than or equal to 0.05 were utilized in the multivariable analysis to identify associated factors of adequate postnatal newborn care.

### Ethical consideration

Ethics approval was not required for this study since the data is secondary and is available publicly. However, we have been given the authorization letter to download the DHS data. More details concerning DHS data and ethical standards are available at http://goo.gl/ny8T6X.

## Results

### Sociodemographic characteristics of the participants

A total weighted sample of 105,904 respondents were used for this analysis. The majority (44.93%) of the respondents were between the age of 15 and 24 with a median age of 25 (IQR = 19–35). About 73,238 (69.15%) of the participants were rural dwellers. Forty-five percent of the study participants were from poor wealth quantiles (Table 2).

**Table 2:** Socio-demographic and other characteristics of respondents in SSA, 2023 (n = 105904).

Variables	Category	Frequency	Percent
Age of respondents in years	15–24	37602	35.51
	25–34	47585	44.93
	≥35	20717	19.56
Residence	Urban	32666	30.85
	Rural	73238	69.15
Sex of household head	Male	83741	79.07
	Female	22163	20.93
Current marital status of respondents	Married	75820	71.59
	Not married	30084	28.41
Educational status of respondents	No education	36141	34.13
	Primary education	38836	36.67
	Secondary education	26975	25.47
	Higher education	3952	3.73
Educational status of partner(n = 91559)	No education	33835	36.95
	Primary education	28022	30.61
	Secondary education	23226	25.37
	Higher education	6475	7.07
Media exposure	No	38879	36.71
	Yes	67025	63.29
Respondents currently working	No	41141	38.85
	Yes	64764	61.15
Wealth status	Poor	47779	45.12
	Middle	21287	20.10
	Rich	36837	34.78
Community-level poverty	Low	50983	48.14
	High	54921	51.86
Community-level media exposure	Low	51043	48.20
	High	54861	51.80
Visit the health facility for the last 12 months	No	34917	32.97
	Yes	70987	67.03
Distance to the health facility	Big problem	41175	38.88
	Not big problem	64729	61.12

### Obstetrics-related characteristics of respondents

About 72,214 (68.19%) of the respondents gave birth in the health facility. Only a small number 6,721 (6.35%) of the respondents had eight or more ANC visits based on the newly recommendation of ANC by WHO (Table 3).

**Table 3:** Obstetric-related and child characteristics of mothers in SSA countries, 2023 (n = 105904).

Variables	Categories	Frequency	Percent
Number of ANC visit	0	12740	12.03
	1–7	86443	81.62
	8+	6721	6.35
Current pregnancy	No	100832	95.21
	Yes	5073	4.79
Number of living children	No	1207	1.14
	1–2	47695	45.04
	3+	57002	53.82
Age of mother at first child in years	<18	34899	32.95
	18+	71006	67.05
Place of delivery	Home	33690	31.81
	Health facility	72214	68.19

### Newborn related characteristics

An approximately equal proportion of the newborns (50.84% male) and (49.16% female) were included in this study; 52.23% were between the ages of 0 and 11 months with a median age of 14 (IQR: 10, 18) months. The majority of the newborns (80.29%) were currently breastfed, and almost all (98.27%) of childbirth were singleton (Table 4).

**Table 4:** Child characteristics in SSA countries, 2023 (n = 105904).

Variables	Category	Frequency	Percent
Sex of child	Male	53843	50.84
	Female	52061	49.16
Current age in months	0–11	55309	52.23
	12–17	27753	26.21
	18–23	22842	21.57
Child is twin	Single	104070	98.27
	Multiple	1835	1.73
Currently breast feed	No	20875	19.71
	Yes	85030	80.29
Birth order	≤3	62094	58.63
	>3	43810	41.37

### Prevalence of adequate content of PNC for the newborn in SSA countries

**Fig 1:**
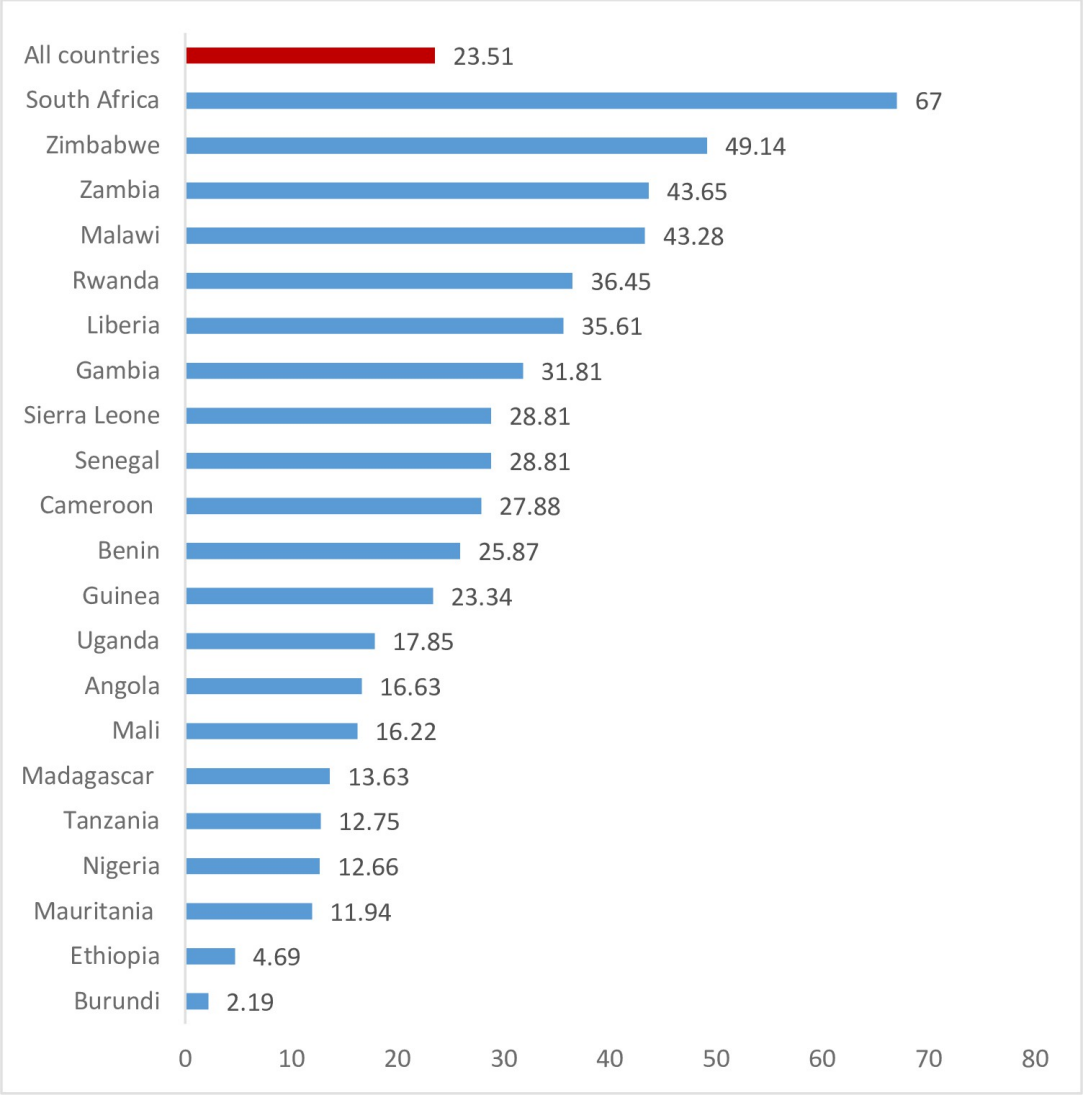
Prevalence of adequate content of postnatal care for the newborn in SSA countries, 2023.

The prevalence of adequate content of PNC for newborns in sub-Saharan Africa was 23.51% (95% CI: 23.26, 23.77). Among those countries, Burundi recorded the lowest adequate content of PNC for newborns (2.19%), and the highest was observed in South Africa (67%) (Fig 1).

### Factors associated with adequate content of PNC for the newborn in SSA countries

In this analysis, a multivariable multilevel model was used to examine the factors associated with adequate content of postnatal care (PNC) for newborns. From the final model, it was found that residence, community-level poverty, and Sub-Region of Sub-Saharan Africa (SSA) were the community-level variables significantly associated with adequate content of PNC for newborns. Similarly, the age of the respondents, level of education of the respondents, wealth status, number of ANC visits, and place of delivery were the individual-level variables significantly associated with adequate content of PNC for the newborn. After adjusting other individual and community-level variables, the odds of adequate content of PNC for the newborn whose mothers aged ≥ 35 were 1.12 (95% CI = 1.06,1.16) times higher compared to those aged 15–24. Newborns whose mothers attended primary, secondary, and higher education had 1.18 (95% CI = 1.13, 1.23)), 1.58 (95% CI = 1.51,1.66), and 1.61 (95% CI = 1.49,1.75) odds of adequate content of PNC, respectively as compared to those who did not have formal education (no education). Newborns from households with rich wealth status had 1.05 (95% CI = 1.01,1.10) times higher odds of adequate content of PNC in contrast to those who were from the poor households. The odds of adequate content of PNC for newborns whose mothers attended ANC 1–7, 8 and above were 1.61 (95% CI = 1.51, 1.73) and 2.54 (95% CI = 2.32, 2.77) times higher, respectively as compared to no antenatal care visits. Newborns who were born at a health facility had 4.37 (4.16,4.58) times higher odds of adequate content of PNC as compared to those born at home.

With regard to the community level factors, the odds of adequate content of PNC for the newborn who lived in East, Central, and West African subregions were decreased by 77% [AOR = 0.23,95% CI = (0.20,0.26)], 79% [AOR = 0.21,95% CI = 0.19,0.24)] and 77% [AOR = 0.23,95% CI = 0.21, 0.27)], respectively relative to Southern African regions. Urban dweller newborns were 1.22 (95% CI = 1.17,1.27) odds of adequate content of PNC compared to those who lived in rural areas. The odds of adequate content of PNC for the newborn who were from low community poverty were 1.21 (95% CI = 1.11,1.31) times higher than their counterparts (Table 5).

**Table 5:** Factors associated with adequate content of postnatal care for the newborn in sub-Saharan Africa countries (n = 105904).

Variables	Null model	Model I AOR (95% CI)	Model II AOR (95% CI)	Model III AOR (95% CI)
**Age of respondents**				
15–24		1		1
25–34		1.01(0.97, 1.04)		0.98(0.95,1.01)
≥35		1.15(1.09, 1.20)		1.12(1.06,1.16)**
**Residence**				
Rural			1	
Urban			1.86(1.79,1.92)	1.22(1.17,1.27)**
**Marital status**				
Not married		1		1
Married		0.97(0.94, 1.01)		1.03(0.99,1.07)
**Educational status**				
No education		1		1
Primary education		1.16(1.11, 1.20)		1.18(1.13,1.23)***
Secondary education		1.69(1.62, 1.77)		1.58(1.51,1.66)***
Higher education		1.76(1.63, 1.91)		1.61(1.49,1.75)***
**Working status of respondents**				
Not working		1		1
Working		0.95(0.92, 0.98)		1.01(0.98,1.04)
**Wealth status**				
Poor		1		1
Middle		0.98(0.94, 1.03)		0.99(0.94,1.02)
Rich		1.07(1.03, 1.12)		1.05(1.01,1.10)*
**Community-level poverty**				
High			1	
Low			1.07(0.99,1.168)	1.21(1.11,1.31)**
**Number of ANC visit**				
0		1		1
1–7		1.54(1.44,1.65)		1.61(1.51,1.73)***
8+		2.53(2.32, 2.77)		2.54(2.32,2.77)***
**Place of delivery**				
Home		1		1
Health facility		4.52(4.31,4.73)		4.37(4.16,4.58)***
**Distance to health facility**				
Big problem			1	
Not big problem			1.11(1.08,1.15)	0.94(0.91,1.00)
**Child sex**				
Female		1		1
Male		0.98(0.95, 1.01)		0.98(0.95,1.01)
**Media Exposure**				
No		1		
Yes		1.07(1.03,1.11)		1.03 (0.99,1.07)
**Community-level Media exposure**				
Low			1	1
High			1.25(1.15,1.36)	1.07(0.99,1.16)
**Sub-Region of sub-Saharan Africa**				
East Africa			0.17(0.15,0.195)	0.23(0.20,0.26)***
West Africa			0.15(0.13,0.16)	0.23(0.21, 0.27)***
Central Africa			0.12(0.11,0.14)	0.21(0.19,0.24)***
Southern Africa			1	1
Intercept	0.29(0.28,0.31)	0.04(0.04,0.05)	1.15 (1.00,1.33)	0.15(0.13, 0.18)
**Measure of variation**				
Community-level variance	0.31(0.27,0.36)	0.27(0.22,0.30)	0.30(0.26,0.35)	0.26(0.23,0.31)
ICC	8.64%	7.36%	8.50%	7.21%
MOR	1.70	1.64	1.68	1.62
PCV	Ref	12.90%	3.22%	16.13%
Log likelihood	-56908.94	-52161.48	-55535.162	-51793.90
Deviance(-2LL)	113817.88	104322.96	111070.324	103587.80

*** P-value < 0.001

** p-value < 0.01

* p-value < 0.05

Null model-contains no explanatory variables; Model I-includes individual-level factors only; Model II-includes community-level factors only; Model III includes both individual-level and community-level factors, AOR-adjusted odds ratio, CI-confidence interval

ICC: Intra Class Correlation Coefficient, MOR: Median Odds Ratio, PCV: Proportional Change in Variance.

## Discussion

The provision of postnatal care significantly reduces the risk of morbidity and mortality for both mothers and children. Postpartum care helps healthcare practitioners recognize and treat postpartum issues. The purpose of this study was to determine the prevalence of adequate content of PNC for newborns and its associated factors in sub-Saharan Africa. The prevalence of adequate content of PNC for the newborn was 23.51% (95% CI: 23.26, 23.77). The observed prevalence of adequate content of PNC for newborns is still too low to achieve the desired reduction in postnatal-related child mortality and morbidity. The prevalence of adequate content of PNC for newborns ranged from 2.19% in Burundi to 67% in South Africa. Specifically, South Africa is the leading adequate content of PNC for newborns followed by Zimbabwe (49.14%), Zambia (43.62%), and Malawi (43.28%). On the other hand, Burundi, Ethiopia, Mauritania, Nigeria, Tanzania, Madagascar, Mali, Angola, and Uganda have below 20% coverage of adequate content of PNC for newborns. Several factors may contribute to this low coverage of adequate content of PNC. All the countries included in this study except South Africa, are essentially low-and-medium income countries (LMICs), and it has been found that healthcare services in these low-resource countries are insufficient, substandard, or non-existent. Evidence have shown that mother and child health care services are underserved in LMICs due to limited infrastructure, low levels of education, low or no enlightenment, and poverty [27–31]. Therefore, the variation in the prevalence of adequate content of postnatal care (PNC) for newborns between countries might be attributed to differences in healthcare infrastructure, access to services, health education, socioeconomic factors, and cultural influences. Countries with stronger healthcare systems, better access, higher health literacy, and fewer socioeconomic disparities tend to have higher rates of adequate PNC. Addressing these factors through improved infrastructure, enhanced access, health education, and socioeconomic interventions can help bridge the gap and improve PNC prevalence. Another probable explanation for the low prevalence of adequate PNC is a cultural practice, which prohibits freshly delivered neonates from being touched by anybody or leaving the house until the 10/12th day after birth [32].

The result of the study showed that residence, community-level poverty, and Sub-Region of sub-Saharan Africa were the community-level variables factors significantly associated with adequate content of PNC for the newborn. Likewise, the age of the respondents, level of education of the respondents, wealth status, number of ANC visits, and place of delivery were the individual-level variables significantly associated with adequate PNC for the newborn.

The odds of adequate content of PNC among newborns whose mothers aged ≥ 35 years were higher in contrast to mothers whose age is between 15–24 years. Similar findings were reported in Malawi and SSA [33, 34]. Possibly, this positive relationship might be explained by the likelihood that PNC service experience may increase with women’s age [32]. This study showed that when women’s educational status increases, so do their chances of having newborns who have access to adequate content of PNC. This study was in agreement with different studies [34–37]. This might be because as women gain power, they will have access to knowledge about the benefits of using PNC services and will be encouraged to use them. This illustrates that education improves health knowledge and behavior [38].

The odds of adequate content of PNC among newborns whose mother lives in urban areas were higher compared to newborns whose mother resides in rural areas. Similar findings was reported from different studies, in Nigeria [37], in Nepal [39]. In rural areas, the disadvantages may include access, cost of services, distance and travel time, opportunity costs of leaving work to attend health facilities, and lack of skilled personnel [40, 41]. The other possible explanation might be that child and maternal health services are concentrated around urban areas than rural areas. This can lead to poor health outcomes in the rural areas. This implies that rural areas should be the focus of attention to improve the utilization of adequate postnatal newborn care. To alleviate the gap of newborn care in rural areas interventions may include strengthening healthcare infrastructure, implementing mobile health services, training and deploying community health workers, and providing transportation support. Additionally, raising health education and awareness, as well as offering financial support, can help bridge the gap and improve access to adequate content of PNC in rural areas.

This study found that both the wealth status of the households and the community-level poverty determine the PNC utilization, thus newborns from a rich household and from low community-level poverty had higher odds of adequate content of PNC, respectively compared to poor households and high community-level poverty. This could be due to the fact that women with a higher wealth index are more likely to be knowledgeable [42], and develop an interest to learn [43]. This implies that increasing postnatal newborn care and improving the household wealth status and decreasing the level of community poverty is necessary. Addressing community-level poverty is crucial for improving the overall well-being of communities and, subsequently, the quality of postnatal care provided to newborns. This requires comprehensive poverty alleviation strategies that focus on improving education, income generation, and access to healthcare services.

The likelihood of newborns receiving adequate content of PNC was found to be lower in the East, Central, and West African sub-regions compared to South Africa. This discrepancy may be attributed to variations in literacy levels between South Africa and other regions. For instance, South Africa has a higher literacy rate, estimated at 94.6% [44], which potentially influences health knowledge and the utilization of PNC services. The higher literacy rate in South Africa may contribute to better awareness and understanding of the importance of PNC, leading to increased utilization and ultimately improved health outcomes for newborns. The other possible difference might be due to the difference in gross domestic product (GDP) [45], which in turn may affect the infrastructure of the country.

According to the findings of this study, there is a significant association between antenatal care (ANC) utilization, facility-based delivery, and the likelihood of getting adequate content of PNC for newborns. This finding is supported by previous research studies [33, 34, 46]. It is could be that mothers who received ANC visits and delivered in health facilities are more likely to receive comprehensive counseling on PNC services, including education on identifying neonatal danger signs. This suggests that ANC and facility-based delivery play a crucial role in promoting optimal PNC practices and improving maternal and newborn health outcomes. Efforts should be made to improve access to antenatal care services and ensure that pregnant women receive comprehensive care throughout their pregnancy. This might be achieved through the expansion of healthcare facilities, particularly in underserved areas, and the training and deployment of skilled healthcare professionals.

Regarding the strengths, we used large data set from 21 SSA countries, which is representative across the countries. Moreover, a robust multilevel modeling technique was employed, accounting for the hierarchical nature of the survey data and yielding results that are more reliable. However, the study is not free of limitations, the survey data may be susceptible to recall bias, as mothers were interviewed about care provided within two days after delivery, even if their baby is currently two years old. Furthermore, due to the cross-sectional nature of the study, it may not establish a clear temporal relationship between the independent and outcome variables. These considerations should be taken into account when interpreting the findings.

## Conclusions

The finding of this study showed that the overall prevalence of adequate content of PNC for a newborn in SSA countries was low. Various factors at both the individual and community level were found to be significantly associated with adequate content of PNC, including the respondents’ age, level of education, wealth status, ANC visits, place of delivery, residence, community-level poverty, and sub-region of SSA. Efforts should be directed towards interventions that address these factors to improve the provision of adequate content of PNC for the newborn. Strategies should focus on increasing access to antenatal care services, particularly for vulnerable populations, such as younger mothers, those with lower education levels, and individuals residing in impoverished communities. Furthermore, future research should explore the root cause for the variation of adequate content of PNC across different regions of Africa. This study can inform the development of targeted interventions and policies aimed at improving PNC outcomes and ultimately reducing newborn morbidity and mortality rates in SSA.

## Abbreviations

AOR: Adjusted Odds Ratio
ANC: Antenatal Care
DHS: Demographic health survey
ICC: Intra-class Correlation Coefficient
MOR: Median Odds Ratio
PNC: Post-natal Care
PCV: Proportional Change in Variance
SSA: Subsahara Africa
WHO: World Health Organization
